# Impact of a Pitanga Leaf Extract to Prevent Lipid Oxidation Processes during Shelf Life of Packaged Pork Burgers: An Untargeted Metabolomic Approach

**DOI:** 10.3390/foods9111668

**Published:** 2020-11-15

**Authors:** Gabriele Rocchetti, Letizia Bernardo, Mirian Pateiro, Francisco J. Barba, Paulo E. S. Munekata, Marco Trevisan, José M. Lorenzo, Luigi Lucini

**Affiliations:** 1 Department for Sustainable Food Process (DiSTAS), Università Cattolica del Sacro Cuore, Via Emilia Parmense 84, 29122 Piacenza, Italy; l_bernardo@libero.it (L.B.); marco.trevisan@unicatt.it (M.T.); luigi.lucini@unicatt.it (L.L.); 2 Centro Tecnológico de la Carne de Galicia, rúa Galicia 4, Parque Tecnológico de Galicia, San Cibrao das Viñas, 32900 Ourense, Spain; mirianpateiro@ceteca.net (M.P.); pmunekata@gmail.com (P.E.S.M.); jmlorenzo@ceteca.net (J.M.L.); 3 Nutrition and Food Science Area, Preventive Medicine and Public Health, Food Science, Toxicology and Forensic Medicine Department, Faculty of Pharmacy, Universitat de València, Avda, Vicent Andrés Estellés, 46100 Burjassot, València, Spain; francisco.barba@uv.es

**Keywords:** *Eugenia uniflora*, food metabolomics, mass spectrometry, lipid oxidation, antioxidants

## Abstract

In this work, the comprehensive metabolomic changes in pork burgers treated with different antioxidants, namely, (a) a control without antioxidants, (b) 200 mg/kg butylated hydroxytoluene (BHT), and (c) 250 mg/kg pitanga leaf extract (PLE, from *Eugenia uniflora* L.), each one packaged under modified atmosphere (80% O_2_ and 20% CO_2_) for 18 days storage at 2 ± 1 °C, were deeply studied. In particular, untargeted metabolomics was used to evaluate the impact of the antioxidant extracts on meat quality. The PLE phytochemical profile revealed a wide variety of antioxidant compounds, such as polyphenols, alkaloids, and terpenoids. Multivariate statistics (both unsupervised and supervised) allowed to observe marked differences in BHT and PLE burgers metabolomic profiles during storage. Most of the differences could be attributed to hexanoylcarnitine, 4-hydroxy-2-nonenal, 6-hydroxypentadecanedioic acid, 9S,11S,15S,20-tetrahydroxy-5Z,13E-prostadienoic acid (20-hydroxy-PGF2a), sativic acid, followed by glycerophospholipids. In addition, significant correlations (*p* < 0.01) were observed between thiobarbituric acid reactive substances and metabolites related to lipid oxidation processes. Therefore, the approach used showed a clear modulation of lipid oxidation, likely promoted by the plant leaf extract, thus confirming the ability of PLE to delay lipid oxidative phenomena during storage.

## 1. Introduction

In the last years, the study of factors potentially affecting meat quality throughout the shelf life are increased [1]. In this regard, lipid and protein oxidation represents the main cause of meat deterioration [2,3]. Lipid oxidation is able to produce undesirable effects in meat, altering above all its organoleptic properties [4,5,6]. Therefore, to counteract oxidative reactions during shelf life, synthetic antioxidants, such as butylated hydroxytoluene (BHT), have been frequently exploited [5]. In particular, there is an interest for finding alternative to synthetic antioxidants, by exploiting antioxidants from different sources [7,8,9,10,11]. Recently, extracts from *Paullinia cupana* Kunth seeds and *Eugenia uniflora* L. (commonly known as pitanga) leaves have been used as alternative to BHT during meat storage [5,6,12,13,14]. Overall, it was demonstrated that the addition of 250 mg/kg of guarana or pitanga extract is able to reduce those phenomena typically affecting meat quality, thus representing an effective alternative to synthetic antioxidants to be used during meat processing and/or storage.

*Pitanga* is a plant hailing from tropical South America east-coast extended in Brazil through the north of Argentina. Pitanga leaves are mainly used in medicine to treat diarrhea, hyperglycemia, hypertension, and hyperlipidemia. In addition, pitanga leaves possess antifungal, antibacterial, and cytotoxic properties [15]. Interestingly, both pulp and leaf are abundant in phenolic compounds, such as flavonoids (including tannins, flavonols, and anthocyanins), followed by terpenoids and carotenoids [5]. Besides, in a recent work, pitanga leaf extracts added to pork burgers demonstrated a strong in vitro antioxidant activity [5]. In addition, Chakravartula et al. [16] recently incorporated a pitanga leaf extract (PLE) to a film based on blends of cassava starch/chitosan, showing no effect of PLE on the film mechanical properties. During meat storage, several processes may occur, affecting the principal meat quality parameters, such as color, which has impact on consumer acceptability [6,17]. In this regard, lipid and protein oxidation results in the production of several off-flavors. Besides, there is an increased risk of spoilage by different microorganisms [4,18]. Therefore, in the last years, different -omics approaches have been used for evaluating both quality and safety of meat, mainly focusing on the deleterious processes occurring during meat processing and/or storage [19]. In addition, as suggested in literature [20], metabolomics has the great potential to identify biomarkers of food spoilage by pathogenic microorganisms, which may facilitate the development of techniques to detect and control microbial growth.

In a previous work [5], we fully characterized the polyphenol composition of pitanga leaf extracts (PLE) added to pork burgers during storage. In particular, PLE was proposed as a valid alternative to BHT in order to extend the shelf life of pork burgers. Therefore, in this work, we used a comprehensive untargeted metabolomic approach, as chemometric tool, to provide new insight into the chemical changes of the same pork burgers added with both BHT and PLE, during storage at 2 ± 1 °C. In fact, to date, scarce information is available on the application of untargeted metabolomics to study meat oxidation during storage. In particular, using untargeted metabolomics could reveal a wider number of discriminant compounds related to oxidation of meat, when compared to targeted or classical approaches, thus highlighting the possible exploitation of PLE by food and meat industry. Such knowledge could be very helpful for meat science area, to provide a comprehensive understanding on the effects of different technological processes and to explain the sensory, nutritional, functionality, and nutraceutical quality of the final product.

## 2. Materials and Methods

### 2.1. Preparation of Pork Burgers

The preparation of pork burgers together with their packaging followed the protocol as detailed by Zamuz et al. [17], with minor modifications. Three treatments were prepared: a control (with no antioxidant added), a sample with 200 mg/kg BHT (BHT), and other with 250 mg/kg of pitanga leaf extract (PLE). The detailed information regarding the PLE used is reported in our previously published work [5]. A total of 90 pork burgers (three treatments × three sampling points × five samples for each sampling point × two different processing batches) were manufactured in the pilot plant of the Meat Technology Center of Galicia. Pork burgers of 100 g (*n* = 5 per batch and storage time) were prepared using the primal cuts of pig shoulder and loin with a fat content between 3% and 6%. The meat was ground through an 8 mm diameter mincing plate in a refrigerated mincer machine (La Minerva, Bologna, Italy), mixed and compressed manually. Burgers were produced in molds of 10 cm diameter and 1 cm height in a burger maker (A-2000, Gaser, Girona, Spain). Pork burgers were packed in 300 mm thick PET-EVOH-PE (Polyethylene terephthalate (PET)-Ethylene vinyl alcohol copolymer (EVOH)-Polyethylene (PE)) trays, which were sealed with multilayer PE-EVOH-PE film 74 mm thick and permeability of 2 mL/(m^2^ bar/day) suitable for gas mixtures (Viduca, Alicante, Spain). The packaging was carried out using a heat sealer (LARI3/Pn T-VG-R-SKIN, Ca.Ve.Co., Palazzolo, Italy), after injection of the gas mixture containing 80% O_2_ and 20% CO_2_. Afterwards, pork burger samples were stored at 2 ± 1 °C under light in order to simulate supermarket conditions and placed in metal shelving, receiving a range of 15–20 of lux values depending on tray position. Finally, metabolomic changes, pH, and lipid oxidation of pork burger samples were analyzed considering three time points of storage, being 0, 11, and 18 days, respectively. In addition, at day 0, a characterization of pork patties was carried out following the methods previously established by Pateiro et al. [21].

### 2.2. Untargeted Metabolomics-Based Analysis of PLE and Pork Burgers during Storage

The untargeted profile of the PLE, fully characterized from a polyphenolic point of view in our previous published paper [5], was further investigated using an untargeted metabolomics. In this regard, the extraction and analysis of metabolites in PLE was carried out following the protocol previously reported [5]. In particular, the compounds annotation was recursively achieved against the comprehensive database FoodDB (http://foodb.ca/) in order to putatively annotate some other compounds characterizing the PLE. Regarding pork burgers, 1 gram of each sample was extracted in 10 mL of an 80% methanolic solution acidified with 0.1% formic acid, using a homogenizer-assisted extraction (Ultra-turrax, Ika T25, Staufen, Germany). The extracts were centrifuged (6000× *g* for 15 min at 4 °C), and then filtered using 0.22 μm cellulose syringe filters. Finally, the filtered solutions were collected in amber vials.

The comprehensive screening of meat metabolites was performed using an untargeted metabolomic approach, based on ultra-high-pressure liquid chromatography (UHPLC) coupled with quadrupole time-of-flight (QTOF) mass spectrometry, as previously reported [22,23,24]. Briefly, a reverse-phase chromatography system was performed, using a BlueOrchid C18 column (100 mm × 2 mm i.d., 1.8 μm; from Knauer, Berlin, Germany), with a gradient of methanol in water (from 6% to 94% in 33 min) as mobile phase. Elution was operated with a flow rate of 0.22 mL/min and the injection sequence was randomized, with five replicates (6 μL injection volume) for each sample, and quality control samples (prepared by pooling same aliquots of each sample) were run every 9 samples. In addition, the instrument acquired ions in positive full scan mode (50–1200 m/z, “extended dynamic range” mode). Raw metabolomics-based data were processed using the software Profinder (version B.06; from Agilent Technologies, Santa Clara, CA, USA), considering the entire isotopic pattern (i.e., monoisotopic accurate mass with an accuracy below 5 ppm, isotope spacing, and isotope ratio) for compound annotation. Thereafter, raw data were aligned and deconvoluted using the Agilent Profinder B.07 software. The find-by-formula algorithm was used to annotate molecular features (MFs) following mass and retention time alignment. The minimum absolute abundance was set to 5000 counts, the mass accuracy was 5 ppm, and the isotope model of “common organic molecules” was adopted. The list of possible molecular formulae was provided by considering their accurate monoisotopic masses (mass error ≤ 5 ppm) and isotopic patterns (i.e., isotopic distribution, space, and abundance). These latter were compared to those reported in the comprehensive FoodDB (https://foodb.ca/). Features were aligned (mass tolerance window: 5 ppm + 2 mDa; retention time tolerance: 0.15 min), and a post-acquisition filtering-by-frequency process was also adopted, to retain features present in 100% of replications within at least one treatment. Overall, in our untargeted experiments, a Level 2 of identification (ID) was achieved (i.e., putatively annotated compounds), as reported by COSMOS Metabolomics Standards Initiative. The Profinder settings used are provided in Table S1. The approach used allowed a great confidence in the annotation process when considering the MS-only conditions.

### 2.3. Statistical Analyses

Metabolomics data were elaborated using the software Mass Profiler Professional (version B.12.06; from Agilent Technologies) as previously reported [24] for data processing and normalization. In this regard, compounds abundance was log2 transformed, normalized at 75th percentile, and then baselined vs. the median of each compound in all sample replicates. Unsupervised hierarchical cluster analysis (HCA; setting the similarity measure as “Euclidean” and “Wards” as the linkage rule) and principal component analysis (PCA) were then used considering the processed MFs, in order to group samples according to intrinsic similarities in their determinations. Thereafter, the metabolomic dataset was exported into SIMCA (version 13; from Umetrics, Malmo, Sweden) software, Pareto scaled, and elaborated using orthogonal projections to latent structures discriminant analysis (OPLS-DA), investigating also the outlier distribution by means of Hotelling’s T2 test. Besides, model parameters (R^2^Y and Q^2^Y) were also inspected. Finally, OPLS-DA cross-validation was done calculating analysis of variance (ANOVA) of the cross-validated (CV) residuals (*p* < 0.01), checking the Hotelling’s T-squared distribution for outliers (using as confidence limits 95% and 99%, for suspect and strong outliers, respectively), and inspecting permutation testing (number of permutations = 200) to exclude model overfitting. The selection method based on variables important in projection (VIP) was then used to list the most important pork metabolites as affected by the storage conditions, while S-plot was performed to combine the modelled covariance and correlation from the OPLS-DA model (considering the comparison PLE and control samples at 18-days of storage). Finally, to achieve information on the increasing or decreasing trends of each discriminant compound, the VIP approach (VIP score > 1) were combined with a volcano plot analysis (Fold-Change analysis with a cut-off value ≥ 2, ANOVA with *p* < 0.05, and Benjamini–Hochberg method to control false discovery rate). This approach was used to gain a better understanding into the changes in oxidation processes occurring over the time points considered. Pearson’s correlation coefficients were then inspected by means of the software SPSS (version 25.0) in order to check correlations between malondialdehyde (MDA) content and the VIP metabolites related to oxidation phenomena. Finally, a misclassification table made in the same software was used to classify the prediction set observations, thus displaying the overall class prediction accuracy.

## 3. Results and Discussion

### 3.1. UHPLC-QTOF-MS Characterization of Pitanga Leaf Extract (PLE)

The leaves of *E. uniflora* have been widely analyzed for their phytochemical composition. In fact, the plant is known to be rich in secondary metabolites such as polyphenols (mainly flavonoids), terpenes, and alkaloids. Interestingly, these compounds are reported to possess several health-promoting properties; in fact, the alkaloids have been correlated with antidiabetic activity [25,26], while *E. uniflora* leaf extracts (i.e., rich in flavonoids, phenolic acids, and alkaloids) have been correlated with antidepressant and antiobesity effects [27]. The chemical composition of PLE has been previously characterized using different analytical platforms, such as liquid chromatography (LC) coupled with diode array detector and mass spectrometry [28,29] and gas chromatography-mass spectrometry (GC-MS) [27]. However, in this work, we used an untargeted metabolomic approach based on UHPLC-QTOF mass spectrometry and the database FoodDB (one of the most comprehensive databases on food constituents) for compounds’ annotation, in order to provide more insights into its phytochemical profile. 

The untargeted UHPLC-QTOF analysis of PLE allowed to putatively annotate 257 compounds according to their accurate mass and isotopic profile. A detailed list of all PLE compounds identified, with the corresponding abundances, composite mass spectra, and ID information, is provided in supplementary material (Table S2). The compounds most representing the metabolomics-based dataset (in terms of relative abundance) were two monoterpenoids (i.e., bornyl diphosphate and isobornyl isobutyrate), three polyphenols (i.e., cinnamic acid, umbelliferone, and caffeic acid), the alkaloid lansiumamide C, and the lysophospholipids LysoPE (0:0/18:2(9Z,12Z)). Overall, the compounds putatively annotated in our experimental conditions were in agreement with the chemical composition recently reported in literature for pitanga leaves (*E. uniflora*), when considering the most important classes [27,30,31]. In fact, PLE is reported to be a source of flavonoids followed by tannins and terpenoids. Accordingly, the most frequent compounds in our PLE belonged to polyphenols (mainly flavonoids and phenolic acids), alkaloids, and terpenoids (Table S2). Among flavonoids, the most representative subclasses were flavonols, anthocyanins, and flavones; additionally, as confirmed in our previous work [5], the untargeted approach allowed us to observe a wider number of low-molecular-weight phenolic acids (mainly belonging to hydroxycinnamic acid and tyrosol derivatives). In a previous work, the analysis of pitanga leaves revealed the presence of alkaloids, nitrogenated derivatives, and some fatty acids [27]. Accordingly, the UHPLC-QTOF-MS results highlighted a high number of alkaloids, fatty acids, and nitrogenated compounds (Table S2). In particular, a great abundance of diglycerides and phospholipids (i.e., glycero- and lysophospholipids), linoleic acid derivatives (such as 18-oxo-oleate), and prenol lipids was observed. Therefore, the results gained from our PLE extracts using untargeted metabolomics provided a wider compounds coverage than targeted approaches [27,30], thus highlighting the rich distribution of antioxidant compounds in this plant matrix. In a previous work [29], a PLE was tested as natural antioxidant for enhancing canola oil stability by monitoring lipid oxidation parameters. The extract was rich in total phenolics (i.e., 229.4 mg gallic acid equivalents/g) with two major phenolic compounds identified, namely, myricitrin and quercetin 3-α-fucopiranoside (both belonging to flavonoids). Overall, the incorporation of PLE was found to decrease primary and secondary lipid oxidation products with respect to the control sample. Accordingly, in our experimental conditions, we found several quercetin derivatives (i.e., dihydroquercetin, quercetin 3-*O*-galactoside, quercetin 4′-*O*-glucoside, and quercetin 3-*O*-xyloside) together with glycosidic forms of myricetin (such as dihydromyricetin 3-*O*-rhamnoside and myricetin 3-*O*-rhamnoside). In addition, caffeic acid was another main constituent of the PLE under investigation. This compound belongs to hydroxycinnamic acids, and it was previously described as one of the most important compounds to enhance the stability of meat products [1], although the antioxidant effects of caffeic acid are strictly dependent by food processing, type of ingredients, and ratio of antioxidants and lipid components [32]. Besides, the antioxidant and prooxidant effects of caffeic acid and its derivatives have been reported through a combination of mechanisms, mainly involving radical scavenging activity, inhibition of lipid peroxidation and shielding against low-density lipoprotein oxidation [32]. Another abundant compound characterizing the metabolomic dataset was umbelliferone, also known as 7-hydroxycoumarin, widely spread in plant kingdom and with recognized antioxidant properties against lipid peroxidation [33]. Regarding other strategies for preventing lipid oxidation, the extracts from green tea (*Camellia sinensis*) are among the most potent antioxidants protecting lipids; however, these extracts were found to induce protein polymerization, due to the formation of covalent protein–phenolic interactions, thus affecting both meat gelation properties and tenderness [34]. In this regard, Jongberg et al. [34] demonstrated that water-soluble caffeic acid derivatives in aqueous extracts of mate (*Ilex paraguariensis*) are able to improve the antioxidative efficiency against thiol oxidation and protein polymerization, as compared to green tea extracts. 

### 3.2. Untargeted Profile and Multivariate Statistical Discrimination of Pork Burgers during Storage

In the last years, untargeted metabolomics has emerged as potent tool to assess the overall quality and safety of foods [35]. In particular, this method is able to provide an impartial and holistic approach and then, using statistical evaluations on the mass features, it makes possible to highlight significant changes between two or more groups. In this work, the untargeted UHPLC-QTOF-MS approach allowed to annotate 1337 MFs. Thereafter, using the comprehensive FoodDB (i.e., the most comprehensive database on food constituents), we putatively annotate 407 meat metabolites. A detailed list containing MFs and the meat metabolites annotated against FoodDB, together with their composite mass spectra, is reported as supplementary material (Tables S3 and S4, respectively). The most represented category of compounds belonged to lipids, such as fatty acyls, glycerophospholipids (mainly lysophospholipids), prenol lipids, organooxygen, and organonitrogen compounds, followed by carboxylic acid and derivatives. Interestingly, the compounds provided by untargeted metabolomics are quite representative of the typical phospholipid’s distribution in meat [36]. In fact, the lipid composition of pork usually includes saturated, monosaturated, and polyunsaturated fatty acids. In addition, there are many other phospholipids that contribute to the taste and aroma of meat that can be divided into glycerophospholipids and sphingolipids, comprising phosphatidylinositol (PI), phosphatidylethanolamine (PE), phosphatidylserine (PS), phosphatidylcholine (PC) and sphingomyelin (SM) subclasses. Interestingly, the untargeted approach allowed to annotate the organonitrogen compound spermine, together with its precursor, namely, spermidine. The presence of spermine is more limited than spermidine; however, only in animal tissues, the content of spermine is comparable to that of its precursor [37]. Another interesting class of compounds characterizing our metabolomic dataset was that of aldehydic compounds, including hexanal, 4-hydroxy-2-hexenal, 2-heptenal, and 4-hydroxy-2-nonenal (Table S4). 

**Figure 1:**
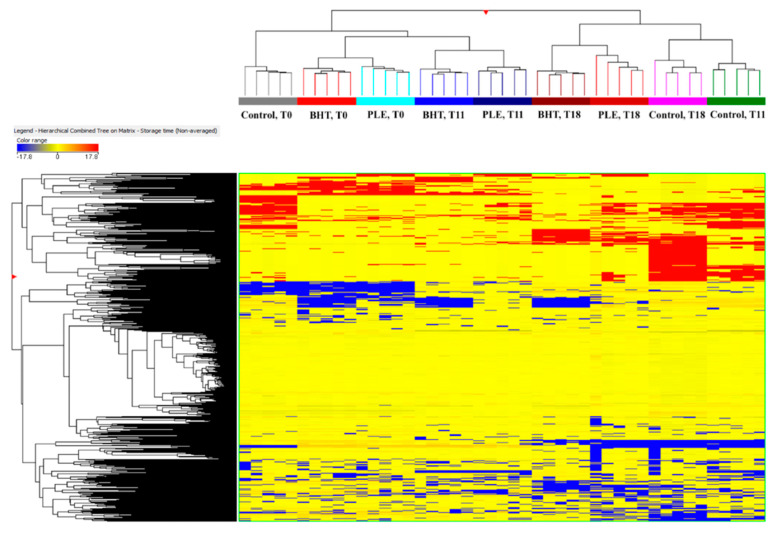
Unsupervised hierarchical cluster analysis (HCA) carried out from the fold-change distribution of each metabolite detected in butylated hydroxytoluene (BHT), pitanga leaf extract (PLE), and control pork samples during storage (i.e., at 0, 11, and 18 days of storage) by using untargeted metabolomics.

Considering the high complexity of the metabolomic dataset (representative of the matrix analyzed), multivariate statistics was then used in order to facilitate the data distribution. First of all, the HCA produced from the fold-change-based heat map on MFs was inspected and reported as Figure 1. 

**Figure 2:**
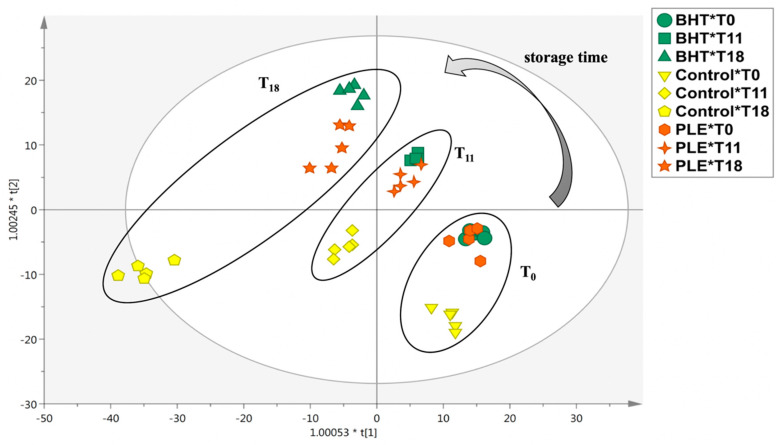
Orthogonal projections to latent structures discriminant analysis (OPLS-DA) considering butylated hydroxytoluene (BHT), pitanga leaf extract (PLE), and control pork samples during storage (i.e., at 0, 11, and 18 days of storage).

As it can be observed, the figure resulted in two main groups: the first cluster included all sample at time-point T0, together with BHT- and PLE-treated samples at 11-days storage (T11), while the second cluster consisted in all samples after 18-days storage (T18) together with the control at T11. The heat-map clearly showed the modification of meat metabolites profile during storage, suggesting also marked differences between control and treated samples mainly after 11 days of storage. Similar information was obtained by inspecting the PCA score plot (Table S5), highlighting a clear difference between control sample (at T11 and T18) and the other samples. In particular, we found a potential effect of both storage time (mainly on control pork sample) and antioxidants addition (BHT and PLE) on the meat metabolomic profile. Thereafter, in order to confirm the trends highlighted by HCA, a following supervised multivariate method, namely, OPLS-DA was applied on the metabolomic dataset. The OPLS-DA score plot was built considering as class membership criterion of the combination of both storage time and matrix analyzed. The resulting output is provided as Figure 2. 

The supervised OPLS-DA model clearly discriminated samples over the storage time points, highlighting a clear difference in the metabolomic profile of control sample. In addition, this was particularly true when considering 18 days of storage (Figure 2). Interestingly, the goodness parameters of the OPLS-DA model were excellent, being R^2^Y = 0.95 and Q^2^Y = 0.67, with adequate statistical significance (*p*-value of cross-validation ANOVA < 0.01; Table S6). Besides, no outliers were observed by inspecting the Hotelling’s T-squared distribution, while the permutation testing excluded overfitting of the OPLS-DA model built (Figure S1). In addition, the misclassification table (Table S6) showed an overall class prediction accuracy of 100% (Fisher’s probability: 1.6 × 10^−32^). Therefore, all these factors confirmed the robustness of the OPLS-DA model built, based on pork burger metabolites during storage. The OPLS-DA score plot showed specific trends during storage according to the different metabolic profiles, likely promoted by the addition of BHT and PLE to pork burgers. In our previous work [5], we found that PLE delayed the discoloration of pork burgers by reducing the loss of redness. Besides, PLE (i.e., 250 mg/kg) was found to promote a decrease in lipid and protein oxidation during the entire storage period, with similar trends to commercial synthetic antioxidant BHT. Therefore, the OPLS-DA score plot on meat metabolomic profiles corroborated the previously reported results, with BHT- and PLE-treated samples clustering together in each time-point considered (Figure 2). On the other hand, control sample was very different when compared to the treated samples, showing a complete separation on the first latent vector [t1] of the OPLS-DA score plot and with the highest variability at T18. 

### 3.3. pH and Lipid Oxidation in Pork Burgers during Storage

Briefly, the chemical composition (in percentage values) of the pork burgers consisted in moisture: 73.69 ± 0.30%, protein: 19.14 ± 0.11%, intramuscular fat: 4.48 ± 0.40%, and ash: 2.24 ± 0.03%. In addition, as clearly showed in our previous work [5] (based on the analyses of the same burgers), the type of treatment and storage time determined a significant effect (*p* < 0.05) on the pH values of pork burgers. In particular, an initial decrease in pH values (*p* < 0.001) was noticed, but after 11 days, the pH remained constant until the end of storage. At 18 days, samples were not significantly different (5.58, 5.61, and 5.62 for control, PLE and BHT samples, respectively) [5]. Besides, according to our previous findings, higher pH values were found in treated samples (i.e., BHT and PLE) both at the beginning and at the end of storage, as also highlighted in similar available works [12,38]. 

Regarding lipid oxidation, it is recognized as one of the major factors affecting meat quality, determining the production of volatile compounds that are responsible of the off-flavors development. In this regard, the effects of oxidation processes on pork flavor are strictly related to the different intramuscular lipids content and composition, as highlighted by Huang et al. [39]. The by-products generated by lipid oxidation can be classified in 2 groups [2]; the first one include hydroperoxides and conjugated dienes (called primary end products), while the second group is characterized by other compounds such as such as isoprostanes, prostaglandin (PG) F2-like compounds, carbonyls (i.e., ketones and aldehydes), furans, and MDA (also called secondary end products). In our experimental conditions, it was evident from both unsupervised and supervised statistical approaches the impact of PLE on pork burgers during storage when compared with the control and, above all, after 18-days of storage. Therefore, in order to explore the differences imposed by PLE on pork burgers, a following variables’ selection method based on VIP (variable importance in projection) approach was used. This approach is able to provide the so-called VIP scores that highlight those compounds better responsible for the hyperspace separation. Table 1 reports the most discriminant compounds highlighted by VIP selection method and organized in classes, together with their prediction score and Fold Change values (as binary logarithm; resulting from volcano plot analysis), when considering control vs. PLE pork samples. 

The VIP metabolites consisted of 96 compounds, being mainly fatty acyls, glycerophospholipids, prenol lipids, steroids, lipid-like molecules, organooxygen compounds, carboxylic acid and derivatives, and other metabolites. Interestingly, among VIP compounds, the 73% of these compounds were found to be up accumulated when considering the pairwise comparison control vs. PLE pork samples (Table 1), thus indicating clear different profiles after 18 days of storage. As can be observed from Table 1, the fatty acyl compounds possessing the higher up accumulation values were hexanoylcarnitine (LogFC = 21.52), sativic acid (LogFC = 20.78), 20-Hydroxy-PGF2a (LogFC = 20.10), 6-hydroxypentadecanedioic acid (LogFC = 18.46), and 4-hydroxy-2-nonenal (LogFC = 11.17). In addition, two glycerophospholipids, namely, LysoPE(16:1(9Z)/0:0) and LPA(18:1(9Z)/0:0), showed high up accumulation values, possessing LogFC values of 19.25 and 18.75, respectively. Thereafter, in order to compare BHT vs. PLE treatments, an OPLS-DA model considering pork samples at 18 days of storage was built and provided in Table S7. Overall, the 65% of VIP compounds was found to be up accumulated in the BHT pork samples, thus confirming similar metabolomic profiles between BHT and PLE pork samples. Interestingly, among the VIP metabolites, we found an up accumulation of both spermine (Log FC = 0.25 and VIP score = 1.13), likely deriving from spermidine and 16-Hydroxy-hexadecanoic acid (Log FC = 0.49 and VIP score = 1.37) in the BHT samples after 18 days of storage.

Overall, the impact of oxidative processes on meat components (such as lipids and proteins) is known to affect its nutritional and functional value, affecting the perceived quality by consumers [2,5]. In particular, the main targets for lipids oxidation are the polyunsaturated fatty acids (PUFA) and phospholipids. Generally, oxidation processes are categorized into nonenzymatic and enzymatic. Nonenzymatic oxidation can be further divided into autoxidation (mediated by free radicals) and photooxidation (promoted by ultraviolet or singlet oxygen). In our experimental conditions, we found a completely different impact of storage conditions on control and treated samples. In fact, as highlighted by the pairwise comparison control vs. PLE samples at 18 days storage, an abundance of discriminant metabolites related to oxidation phenomena has been highlighted. Interestingly, as reported in Table 1, an up accumulation trend for 4-hydroxy-2-nonenal (HNE) was noticed in control when compared with PLE sample. This aldehydic compound results from oxidation of n-6 polyunsaturated fatty acyls, including some hydroperoxyl peroxides as intermediates (i.e., 9/13-hydroperoxyoctadecadienoate and/or 11/15-hydroperoxyeicosatetraenoate) [40]. Besides, other compounds strictly related to lipid oxidation processes have been highlighted (Table 1). In fact, some primary end products were observed in control sample, such as 8R-hydroperoxylinoleic acid, 6-hydroxypentadecanedioic acid, 16-hydroxy-hexadecanoic acid, 18-hydroxyoleate, and sativic acid. Also, the untargeted approach allowed to observe an up accumulation of a secondary end product (i.e., isoprostanes), namely, prostaglandin F1a and 20-Hydroxy-PGF2a (LogFC of 0.67 and 20.11, respectively). Isoprostanes are prostaglandin F-like compounds (formed via actions of cyclooxygenase) that are produced as direct consequence of lipid oxidation processes. These compounds have been widely considered biomarkers of lipid peroxidation, considering their mechanism of formation (i.e., nonenzymatic oxidation of arachidonic acid) [2]. The corresponding S-plot following OPLS-DA model was produced in order to highlight the potential candidate biomarkers for the comparison control vs. PLE pork samples at 18 days of storage (Table S8). In particular, the coordinates in the lower-left quadrant were metabolites significantly increased in control group compared with PLE T18 group, while those in the upper-right quadrant represent the decreased ones. Overall, among the 24 potential biomarkers significantly increased in control samples, we found an abundance of the same compounds previously described as related to oxidation phenomena, such as 18-hydroxyoleate, 8R-hydroperoxylinoleic acid, 3,4-dimethyl-5-pentyl-2-furanheptanoic acid, and 16-hydroxy-hexadecanoic acid. Therefore, our findings seem to suggest a clear oxidative stress in control sample as promoted by the storage conditions and particularly, higher after 18 days. It is also important to underline that the metabolomic changes observed during storage could be caused by oxidation phenomena. In fact, as PLE include a wide variety of bioactive compounds, changes cannot only be attributed to oxidation but also to other factors, such as microbial degradation. In this regard, besides preventing lipid oxidation, PLEs have shown potential to prolong shelf-life through a bacteriostatic effect. In fact, in our previous work [5], the total counts of bacteria indicated that different dosage of PLE resulted in significant lower microbial counts, mainly at the end of the shelf-life, when compared to control and BHT treatments. In addition, PLE was found to possess antimicrobial activity mainly against Gram-negative bacteria, such as *Salmonella* spp. and *P. aeruginosa*, thus highlighting its great potential for meat application as these microorganisms represent food safety and stability issues in such kind of products [5].

### 3.4. Correlation between Metabolomic Data and MDA Content

The production of aldehydic compounds from oxidative processes in meat is generally assessed by evaluating the thiobarbituric acid reactive substances (TBARS) content, including MDA [5,6]. The results on TBARS assay are reported in Table S9. As can be observed, control samples were unacceptable at 11 days of storage, exciding also the threshold of 0.5 mg/kg related to consumer detection of rancid flavor in meat [41]. Thereafter, in order to confirm the results outlined by untargeted metabolomics, the MDA content at T18 was correlated with the relative abundance of each lipid oxidation metabolite detected (Table 1; compounds in bold). In this regard, the Pearson’s correlation coefficients are reported in Table 2. 

Overall, the MDA content was significantly correlated (*p* < 0.01) with each discriminant metabolite detected. Therefore, these findings confirmed also the validity of the potential discriminant metabolites related to oxidation phenomena, as resulted by S-plot following OPLS-DA. In particular, the highest correlation coefficients were found with four compounds, namely, sativic acid (9,10,12,13-tetrahydroxyoctadecanoic acid), 6-hydroxypentadecanedioic acid, 16-hydroxy-hexadecanoic acid, and 18-hydroxyoleate (Table 2). Regarding the TBARS content (Table S9), it recorded a 2-fold increase in the control sample (from 1.5 at T11 up to 3.9 at T18), while remaining almost similar for BHT- and PLE-treated samples (on average 0.12 at T11 and 0.21 at T18). Therefore, untargeted metabolomics allowed to confirm that PLE could be considered an effective alternative to synthetic antioxidants (e.g., BHT) to extend the shelf-life of pork burgers, improving also its perceived quality.

In the last years, metabolomics emerged as useful tool to cope with the most challenging analytical issues, involving among the others the correlations between metabolite composition and food quality. In particular, the most recent works focusing on meat metabolomics evaluated the distribution of food contaminants (such as antibiotics and veterinary drug) and the detection of biomarkers related to adulteration practices. Overall, our work represents one of the first applications of untargeted metabolomics based on UHPLC-QTOF-MS to find possible correlations between natural products added to meat and meat deterioration (as resulted by oxidation and spoilage phenomena), thus extending previously available information. This topic deserves further investigations considering the possible implications on both human health and meat quality. However, as recently reviewed by Domínguez et al. [4], the wide variety of factors that influence oxidation, different compositions of meat, as well as the complexity of reactions and interactions during lipid oxidation make it practically impossible to develop a unique technique to measure the extent of oxidation processes. In this regard, Ceribeli et al. [42] demonstrated that the modulation of oxidation in meat is an overall process that comprises the contribution of lipids, proteins, and polar metabolites, strictly connected with each other. In addition, in this work, we used untargeted metabolomics, thus providing a relative quantification of each discriminant metabolite, where its spectral pattern and intensity is recorded, statistically compared and used to identify those relevant spectral features that distinguish sample class. Therefore, untargeted metabolomics has the potential to provide a panoramic view covering both primary and secondary metabolites in the selected matrix. In a next future, standardization of the quantitative metabolomics-based workflows will be essential to obtain more accurate information. Taken together, our findings highlighted the suitability of untargeted metabolomics to assess the chemical changes occurring during shelf-life of packaged meat. However, further ad hoc targeted studies aimed to validate and/or extend the discriminant compounds related to meat oxidation and preservation are required.

## 4. Conclusions

In this work, natural antioxidants from pitanga leaves have been used as alternative to synthetic antioxidants to check the development of lipid oxidation phenomena. In particular, the pitanga leaf extracts (PLE) added to pork burgers induced important metabolomic changes, likely due to the abundance of different compounds, such as terpenoids, polyphenols, and alkaloids. The marked changes observed at untargeted metabolomic level in all samples (i.e., control, BHT, and PLE) indicated the impact of antioxidants on the composition of packaged pork patties during the 18 days of storage. Regarding the main changes during storage, multivariate statistics following UHPLC-QTOF-MS allowed to highlight several metabolites related to lipid oxidation phenomena. In particular, these compounds were found to be all up accumulated in the control sample when compared with BHT- and PLE-treated samples. Among the most discriminant compounds, we observed specific metabolites related to the oxidation phenomena, such as hexanoylcarnitine, 4-hydroxy-2-nonenal, 6-hydroxypentadecanedioic acid, 20-hydroxy-PGF2a, and sativic acid, followed by glycerophospholipids and other compounds. Overall, as the utilization of plant extracts becomes to achieve industrial level, it is important to combine them with other healthier strategies such as reduction in or replacement of fat, salt, and nitrite. Besides, the use of innovative processing technologies should be aligned with the production of meat and production by sustainable actions. Looking to our findings, although further studies are still needed (based on the careful assessment of PLE toxicity as well as detailed sensorial analyses), our findings demonstrate a clear impact of the plant extracts on the oxidation phenomena. This preliminary study extends the few information available in literature about the use of untargeted metabolomics as potential tool to assess the main chemical changes during shelf life of meat products. Therefore, the search for effective and practicable solutions implementing these extracts in active packaging are advisable and might find huge interest in the future.

## Figures and Tables

**Table 1:** Variable importance in projection (VIP) scores (together with their standard error) following orthogonal projections to latent structures discriminant analysis (OPLS-DA) modelling and fold-change (FC) values (resulting from volcano plot analysis) considering the comparison control vs. pitanga leaf extract (PLE) samples during 18 days of storage. Compounds highlighted in bold are related to lipid oxidation processes.

Classification	Discriminant Compounds	VIP Scores	LogFC (Control vs. PLE)T_18_	Accumulation
Fatty acyls	Cetyl alcohol	1.37 ± 0.59	−1.77	Down
	Isobutyryl-L-carnitine	1.34 ± 0.44	−0.25	Down
	Avocadene 2-acetate	1.29 ± 0.72	−1.37	Down
	7,8-Dihydrovomifoliol 9-(apiosyl-(1->6)-glucoside)	1.15 ± 0.84	−0.47	Down
	Docosenic acid	1.10 ± 0.90	−0.93	Down
	L-Acetylcarnitine	1.09 ± 0.65	−1.10	Down
	Sodium oleate	1.06 ± 0.47	−0.34	Down
	Stearoylcarnitine	1.01 ± 1.08	−0.18	Down
	Methyl 2E,4Z-hexadecadienoate	1.36 ± 0.62	0.14	Up
	2-Hexenyl octanoate	1.02 ± 0.90	0.18	Up
	2-Octenoylcarnitine	1.41 ± 0.77	0.57	Up
	Prostaglandin F1a	1.05 ± 0.95	0.67	Up
	Sorbitan palmitate	1.49 ± 0.14	0.67	Up
	Hept-2-en-1-yl isovalerate	1.29 ± 0.65	0.92	Up
	**18-hydroxyoleate**	1.42 ± 0.47	0.93	Up
	**3,4-Dimethyl-5-pentyl-2-furanheptanoic acid**	1.32 ± 0.60	1.24	Up
	MG(0:0/18:2(9Z,12Z)/0:0)	1.43 ± 0.34	1.37	Up
	(9Z)-12-oxo-dodec-9-enoate	1.49 ± 0.26	1.50	Up
	(Z)-15-Oxo-11-eicosenoic acid	1.50 ± 0.26	1.56	Up
	**16-Hydroxy-hexadecanoic acid**	1.52 ± 0.28	2.54	Up
	(9S,10E,12S,13S)-9,12,13-Trihydroxy-10-octadecenoic acid	1.51 ± 0.33	2.67	Up
	**4-Hydroxy-2-nonenal**	1.03 ± 0.85	11.1	Up
	**6-Hydroxypentadecanedioic acid**	1.55 ± 0.22	18.46	Up
	**20-Hydroxy-PGF2a**	1.49 ± 0.24	20.11	Up
	**Sativic acid (9,10,12,13-tetrahydroxyoctadecanoic acid)**	1.60 ± 0.41	20.79	Up
	Hexanoylcarnitine	1.52 ± 0.18	21.52	Up
Glycerophospholipids	PS(14:0/14:1(9Z))	1.14 ± 1.34	−1.66	Down
	LysoPC(22:5(7Z,10Z,13Z,16Z,19Z))	1.02 ± 0.96	−0.25	Down
	LysoPC(20:4(5Z,8Z,11Z,14Z))	1.40 ± 0.46	0.09	Up
	LysoPC(18:2(9Z,12Z))	1.47 ± 0.29	0.32	Up
	PGP(16:0/16:0)	1.42 ± 0.57	0.54	Up
	LysoPC(P-16:0)	1.51 ± 0.42	0.56	Up
	LysoPE(16:0/0:0)	1.54 ± 0.26	0.86	Up
	LysoPC(20:2(11Z,14Z))	1.48 ± 0.31	0.87	Up
	LysoPE(0:0/18:2(9Z,12Z))	1.54 ± 0.13	1.30	Up
	LysoPC(16:0)	1.55 ± 0.15	1.31	Up
	Lysolecithin	1.49 ± 0.31	1.46	Up
	LysoPE(20:0/0:0)	1.51 ± 0.31	1.53	Up
	LysoPE(0:0/18:1(9Z))	1.52 ± 0.15	1.54	Up
	LysoPC(P-18:0)	1.18 ± 0.90	1.65	Up
	LysoPC(16:1(9Z))	1.49 ± 0.26	1.77	Up
	LysoPC(14:0)	1.53 ± 0.05	2.37	Up
	LysoPE(16:1(9Z)/0:0)	1.52 ± 0.15	18.75	Up
	LPA(18:1(9Z)/0:0)	1.43 ± 0.39	19.25	Up
Prenol lipids	R1-Barrigenol	1.56 ± 0.33	−20.26	Down
	Ganodermic acid Jb	1.46 ± 0.61	−20.06	Down
	Melleolide B	1.14 ± 0.85	−0.01	Down
	(1beta,2alpha,3alpha)-1,2,3,24-Tetrahydroxy-12-oleanen-28-oic acid	1.12 ± 0.79	−0.07	Down
	Geranyl benzoate	1.09 ± 1.23	−0.02	Down
	Austroinulin	1.08 ± 0.43	−0.15	Down
	Cichorioside M	1.08 ± 0.73	−0.82	Down
	Prephytoene diphosphate	1.35 ± 0.72	−18.03	Down
	(-)-Isoxanthochymol	1.33 ± 0.63	0.23	Up
	Madlongiside C	1.33 ± 0.68	0.51	Up
	Hovenidulcigenin B	1.53 ± 0.23	0.67	Up
	Hovenidulcigenin A	1.53 ± 0.27	0.91	Up
	Fasciculol C	1.42 ± 0.92	1.25	Up
	Hydroxysintaxanthin 5,6-epoxide	1.19 ± 0.72	1.84	Up
	Avenestergenin B2	1.54 ± 0.25	18.01	Up
Steroids	3-Sulfodeoxycholic acid	1.03 ± 0.76	−0.09	Down
	23-O-beta-D-Glucopyranosyl-25-methyldolichosterone	1.01 ± 1.31	−1.42	Down
	(24R)-5b,8b-Epidioxyergosta-6,22E-dien-3b-ol 3-glucoside	1.41 ± 0.55	0.34	Up
	(3alpha,5beta,7alpha)-23-Carboxy-7-hydroxy-24-norcholan-3-yl-beta-D-glucopyranosiduronic acid	1.23 ± 0.70	0.42	Up
	Notoginsenoside R10	1.48 ± 0.29	1.87	Up
	28-Homobrassinolide	1.53 ± 0.22	2.02	Up
	Lithocholate 3-O-glucuronide	1.50 ± 0.27	2.44	Up
	(3beta,22E,24R)-Ergosta-4,6,8(14),22-tetraen-3-ol	1.53 ± 0.20	18.83	Up
Lipids and lipid-like molecules	PE(P-16:0e/0:0)	1.22 ± 0.66	−0.01	Down
	2-Arachidonylglycerol	1.27 ± 0.49	0.25	Up
	LysoPE(20:4(5Z,8Z,11Z,14Z)/0:0)	1.48 ± 0.36	0.58	Up
	10-Nitrolinoleic acid	1.53 ± 0.32	1.26	Up
	**8R-Hydroperoxylinoleic acid**	1.37 ± 0.57	1.41	Up
Organooxygen compounds	2-Hexadecanone	1.53 ± 0.43	2.77	Up
	Dodecanal dimethyl acetal	1.44 ± 0.62	3.23	Up
	Lupulone	1.48 ± 0.58	18.90	Up
	Dihydrojasmone	1.53 ± 0.14	19.32	Up
	Zingiberol	1.49 ± 0.20	19.40	Up
	Colupulone	1.55 ± 0.20	21.35	Up
Carboxylic acid and derivatives	Racemethionine	1.10 ± 0.79	−0.77	Down
	N-gamma-L-Glutamyl-L-isoleucine	1.19 ± 0.83	0.19	Up
	Desmosine	1.52 ± 0.20	1.78	Up
	Frangulanine	1.54 ± 0.07	2.22	Up
	Sakacin A	1.49 ± 0.15	3.45	Up
	Merodesmosine	1.52 ± 0.14	19.65	Up
Other compounds	Subaphylline	1.31 ± 0.60	0.15	Up
	Arenaine	1.04 ± 1.18	−0.02	Down
	Rotundine C	1.49 ± 0.18	19.24	Up
	(+/-)-2-(5-Methyl-5-vinyltetrahydrofuran-2-yl)propionaldehyde	1.47 ± 0.39	1.35	Up
	(+)-1,2-Epoxyneomenthyl acetate	1.52 ± 0.26	2.22	Up
	protoporphyrin IX	1.54 ± 0.18	19.20	Up
	2-Aminomuconic acid semialdehyde	1.28 ± 0.74	−1.15	Down
	L-Tryptophan	1.14 ± 0.90	−0.69	Down
	3-Hydroxy-11Z-octadecenoylcarnitine	1.35 ± 0.71	0.81	Up
	**3-Methylbutyl 3-oxobutanoate**	1.43 ± 0.38	19.83	Up
	Hesperaline	1.43 ± 0.46	0.76	Up
	Triethanolamine	1.00 ± 1.21	−0.71	Down

**Table 2:** Pearson’s correlations coefficient between malonaldehyde (MDA) content and VIP discriminant metabolites related to lipid oxidation phenomena.

VIP Discriminant Compounds	Correlations with TBARS
18-hydroxyoleate	0.94 **
3,4-dimethyl-5-pentyl-2-furanheptanoic acid	0.78 **
8R-hydroperoxylinoleic acid	0.83 **
16-hydroxy-hexadecanoic acid	0.96 **
4-hydroxy-2-nonenal	0.74 **
6-hydroxypentadecanedioic acid	0.98 **
20-hydroxy-PGF2a	0.93 **
9,10,12,13-tetrahydroxyoctadecanoic acid	0.98 **
3-methylbutyl 3-oxobutanoate	0.91 **

** = *p* < 0.01.

## Supplementary Materials

The following are available online at https://www.mdpi.com/2304-8158/9/11/1668/s1, Table S1: Profinder settings used during the processing workflow. Table S2: Comprehensive metabolomics dataset containing all the compounds annotated in pitanga leaf extract (PLE), together with the corresponding composite mass spectra (mass and abundance combinations). Table S3: List of mass features obtained from UHPLC-QTOF analysis, used to build unsupervised statistical models (i.e., hierarchical clustering and principal component analysis). Table S4: Comprehensive metabolomics dataset containing all the compounds annotated pork burgers during storage using Food Database, together with the corresponding composite mass spectra (mass and abundance combinations). Table S5: Principal component analysis (PCA) considering the molecular features from UHPLC-QTOF analysis, together with the distribution of pooled quality control samples. Table S6: Cross-validation ANOVA parameters and misclassification table following orthogonal projections to latent structures discriminant analysis (OPLS-DA) supervised modelling. Table S7: The important in projection (VIP) discriminant compounds of the comparison butylated hydroxytoluene (BHT) vs. PLE samples at 18 days of storage resulting from OPLS-DA supervised modelling. Table S8: S-plot together with discriminant compounds following OPLS-DA supervised modelling. Table S9: TBARS values of pork burgers during storage at 2 ± 1 °C. Results are reported as mean ± standard deviation (*n* = 5) and expressed as milligram malonaldehyde per kilogram (mg MDA/kg). Superscript letters within each row indicate homogeneous subclasses as resulted from ANOVA (*p* < 0.05; Duncan’s post hoc test). Figure S1: Validation of orthogonal projections to latent structures discriminant analysis (OPLS-DA) discriminant model on pork metabolites profile, as a function of storage time. The outcome of permutation test (*n* = 200) is given in the upper panel (A), whereas Hotelling’s T2 using 95% and 99% confidence limits is given in the lower one (B).
