# Supplementary figures and images for: Impact of a Pitanga Leaf Extract to Prevent Lipid Oxidation Processes during Shelf Life of Packaged Pork Burgers: An Untargeted Metabolomic Approach
Source: Foods. 2020 Nov 15;9(11):1668. doi: 10.3390/foods9111668 (PMC7696221; doi:10.3390/foods9111668)

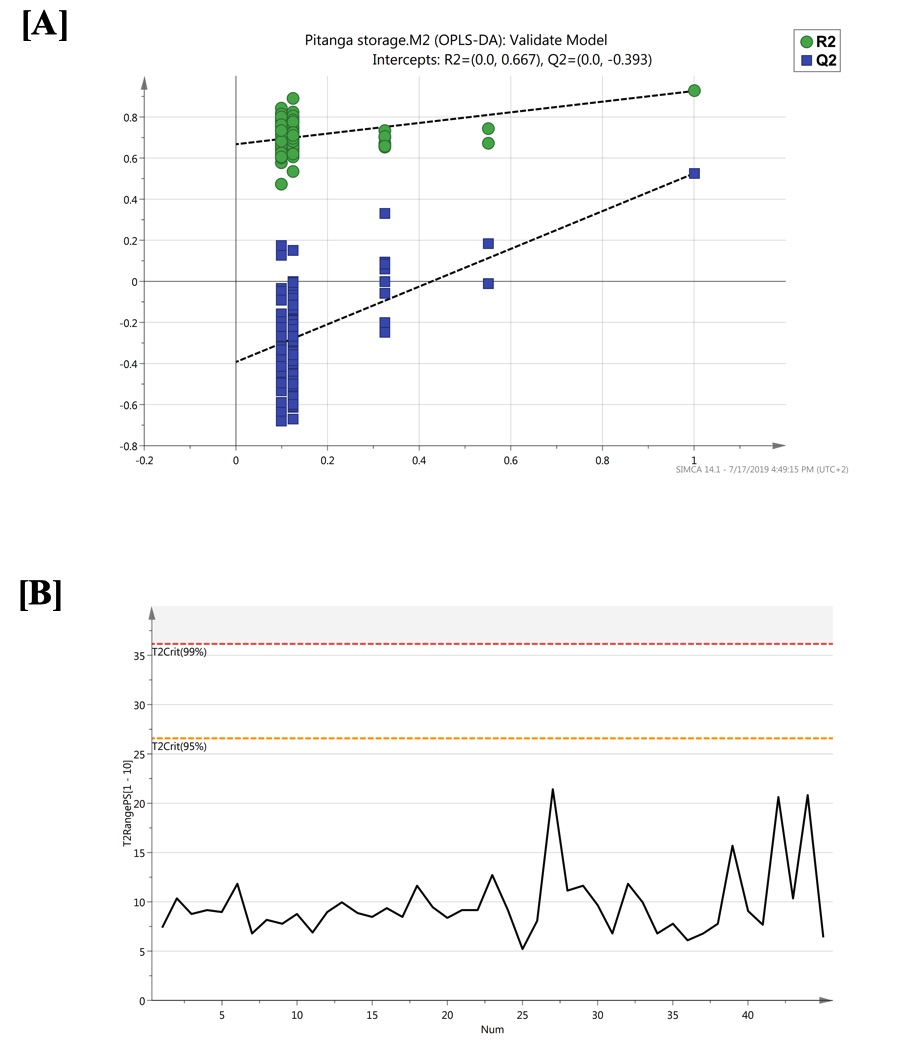

Supplement: Supplementary file 1 [file foods-09-01668-s001.zip › foods-934250 - supplementary Figure S1.jpg]
